# Synergistic Effect of Mandarin Peels and Hesperidin with Sodium Nitrite against Some Food Pathogen Microbes

**DOI:** 10.3390/molecules26113186

**Published:** 2021-05-26

**Authors:** Gouda H. Attia, Diaa A. Marrez, Mona A. Mohammed, Hassan A. Albarqi, Ammar M. Ibrahim, Mohamed A. El Raey

**Affiliations:** 1 Department of Pharmacognosy, College of Pharmacy, Najran University, Najran 1988, Saudi Arabia; 2 Food Toxicology and Contaminants Department, National Research Centre, Cairo 12622, Egypt; diaamm80@hotmail.com; 3 Department of Medicinal and Aromatic Plants Research, National Research Centre, Cairo 12622, Egypt; monaarafamohammed@yahoo.com; 4 Department of Pharmaceutics, College of Pharmacy, Najran University, Najran 1988, Saudi Arabia; haalbarqi@nu.edu.sa; 5 Applied Medical Sciences College, Najran University, Najran 1988, Saudi Arabia; ammar721@yahoo.com; 6 Department of Phytochemistry and Plant Systematics, Pharmaceutical Division, National Research Centre, Cairo 12622, Egypt

**Keywords:** mandarin peel, food preservatives, natural antimicrobials, sodium nitrites

## Abstract

Food preservatives such as NaNO_2_, which are widely used in human food products, undoubtedly affect, to some extent, human organs and health. For this reason, there is a need to reduce the hazards of these chemical preservatives, by replacing them with safe natural bio-preservatives, or adding them to synthetic ones, which provides synergistic and additive effects. The Citrus genus provides a rich source of such bio-preservatives, in addition to the availability of the genus and the low price of citrus fruit crops. In this study, we identify the most abundant flavonoids in citrus fruits (hesperidin) from the polar extract of mandarin peels (agro-waste) by using spectroscopic techniques, as well as limonene from the non-polar portion using GC techniques. Then, we explore the synergistic and additive effects of hesperidin from total mandarin extract with widely used NaNO_2_ to create a chemical preservative in food products. The results are promising and show a significant synergistic and additive activity. The combination of mandarin peel extract with NaNO_2_ had synergistic antibacterial activity against *B*. *cereus*, *Staph*. *aureus*, *E*. *coli*, and *P*. *aeruginosa*, while hesperidin showed a synergistic effect against *B*. *cereus* and *P*. *aeruginosa* and an additive effect against *Staph*. *aureus* and *E*. *coli*. These results refer to the ability of reducing the concentration of NaNO_2_ and replacing it with a safe natural bio-preservative such as hesperidin from total mandarin extract. Moreover, this led to gaining benefits from their biological and nutritive values.

## 1. Introduction

Food preservatives, which are widely used in food products, affect human health. Their effect varies according to age and health status. The most used preservatives are chemicals such as sodium nitrite, NaNO_2_, which is used in meats and fish as an antimicrobial and preservative. Unfortunately, despite its powerful preservative efficiency, NaNO_2_ has various worrisome hazardous effects on human health and safety [1]. In the stomach, NaNO_2_ produces nitrosamines or free radicals. Such products can increase lipid peroxidation, which can pose many health hazards to various body organs. Moreover, reactive nitrogen species have many toxic effects including hepatotoxicity, nephrotoxicity, and dysregulation of inflammatory responses [1].

To minimize or prevent the side effects of these synthetic preservatives, and to enhance the synergistic effect of these synthetic preservatives and natural extracts against foodborne microorganisms, it is essential to use natural food preservatives (bio-preservatives) or multiple combination systems between synthetic food preservatives and natural extracts [2,3]. Natural preservatives are believed to be healthier and have additional benefits due to their bioactivity and nutritional value. That is why a gradual shift from chemical additives to natural alternatives is required [4].

In recent years, the use of natural antimicrobials in the food industry has gained close attention by consumers and producers [5]. The plant kingdom is rich in plants that possess antimicrobial activity. This activity is related to the presence of polyphenols as they contain hydroxyl groups; these groups can interact with the cell membrane of bacteria to disrupt membrane structures and cause the leakage of cellular components [6,7]. In addition to the antimicrobial activity of polyphenols, essential oils have been reported to have antimicrobial activity, and their activity relationship was mentioned [8]. Natural products from plants have diverse biological activities [9,10]. There were 140 medicinal plants screened for their antimicrobial activity including garlic (*Allium sativum*), clove (*Syzygium aromaticum*), *Feijoa sellowiana,* and tee tree (*Melaleuca alternifolia*), and these were described as broad-spectrum antimicrobial agents. Additionally, bearberry (*Arctostaphylos uva-ursi*) and cranberry juice (*Vaccinium macrocarpon*) were reported to treat urinary tract infections [9,10,11].

The Citrus genus, one of the major fruit crops worldwide and a natural source for bio-preservatives, annually produces approximately 133 million tons of the world’s fruit crop, with grapefruit (8.4 million tons), lemons and limes (17.3 million tons), mandarins and tangerines (32.8 million tons), and oranges (73.2 million tons) [12].

The food and industrial processing of citrus peels generate a huge amount of peels as a by-product. Citrus peels are rich in bioactive components such as the hesperidin flavonoid and the limonene monoterpene [12].

Since limonene is commonly recognized as a safe (GRAS) material, it can be used as a food preservative as well as a common food additive to impart a citrus flavor. Limonene has also been tested as an antimicrobial agent, as well as an antioxidant [13].

Flavonoids are important in the defense against pathogenic microorganisms, and they can also be used as natural food preservatives due to their antimicrobial properties. Hesperidin, for example, has anti-infective and anti-replicative properties against many microorganisms [14,15].

Food researchers and the food industry are motivated to produce innovative, natural, and more efficient products as a result of today’s customer preference for natural preservatives over synthetic additives. Along with the current ones, there are undoubtedly many more natural additive ingredients yet to be identified. More research is required to discover new natural preservatives and investigate their safety [8,16].

Mandarin is a cheap fruit crop which is available and highly consumable worldwide. It produces tons of peels (agro-waste) rich in bioactive flavonoids such as hesperidin; therefore, in this work, we selected mandarin to explore the antimicrobial activity of the total extract and the major flavonoid hesperidin with NaNO_2_. The results exhibit significant synergistic and additive effects of both the total extract and hesperidin as antimicrobials.

## 2. Results and Discussion

### 2.1. Identification of Mandarin Oil by GC/MS

**Figure 1:**
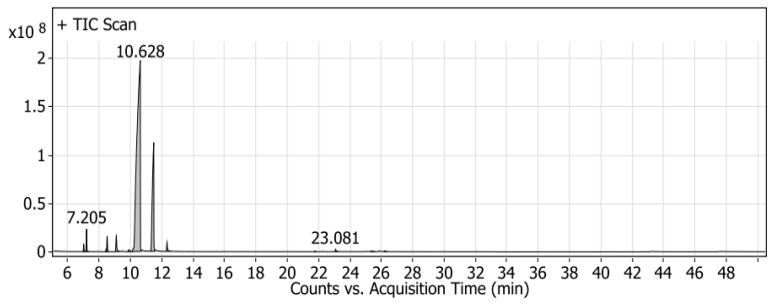
GC/MS chromatogram of mandarin volatile oils.

GC/MS was used to investigate the volatile compounds of the mandarin peel oil produced in Egypt (Figure 1). Thirteen compounds were detected and identified in mandarin peels by comparing the fragmentation pattern with Wiley and NIST Mass Spectral Library data (Table 1). These identified compounds contained two major compounds, namely, limonene (75.21%) and γ-terpinene (18.64%), in addition to β-myrcene (1.53%), α-pinene (1.37%), and 2-.β-pinene (1.13%). The rest of the minor compounds, present at 2.12%, are listed in Table 1.

According to the literature, the percent of limonene in mandarin peels extracted by different methods ranges from 68.51% to 77.59%, and the percent of ɤ-Terpinene ranges from 13.13% to 20.70% [17], which supports our method of extraction and results.

Occasionally, limonene was reported for its food preservative activity. The antibacterial activity of limonene is attributed to its ability to incorporate itself into the bacterial cell membrane lipid system and, hence, alter its properties [18].

### 2.2. Identification of Mandarin Peel Chemical Constituents by UPLC/MS/MS

Formulated mass spectral fragmentations of the metabolites were performed to provide a comprehensive fragmentation pattern with the retention time and MS/MS information. The freeze-dried mandarin ethanol extract MS/MS spectra imported from raw MS data to MS-DIAL 4.36 (http://prime.psc.riken.jp/) (Accessed Date: 18 February 2021) and from Global Natural Products Social Molecular Networking (GNPS, https://gnps.ucsd.edu/) (Accessed Date: 22 February 2021) were used for the identification of mandarin peels by comparing their fragmentation patterns and retention indices (RI) with available databases.

**Figure 2:**
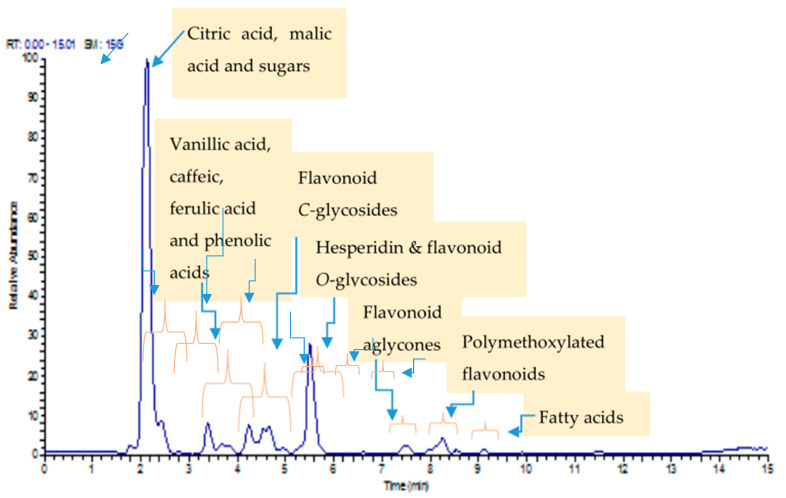
UPLC/MS/MS of mandarin ethanol extract (base peak) chromatogram.

UPLC/MS/MS analysis of mandarin peels led to the identification of 33 compounds (Table 2). These compounds are mainly sugars, flavonoid glycosides, and organic acids, as well as a small amount of methoxylated flavonoids and lipids (Figure 2).

These compounds were dereplicated using GNPS and MS-dial and compared to literature data.

Identification of Sugars

The sugar contents seem to be high in mandarin peels, as shown in Figure 2. We identified two main sugars based on their molecular formula and fragmentation as well as their dereplication with GNPS. These compounds were identified as trehalose (C_12_H_22_O_11_) and hexose (C_6_H_12_O_6_).

It was surprisingly reported that identified sugar trehalose in the presence of fatty acids reduces the adhesion of microbial pathogens to the surfaces [35].

Identification of Organic and Fatty Acids

Two types of organic acids were detected in the mandarin peel ethanolic extract. Two organic hydroxy acids were detected and annotated according to their molecular formula, fragmentation pattern, and retention times, namely, citric acid with molecular ion [M − H]^−^ at *m/z* 191 (C_7_H_12_O_6_) and malic acid with [M − H]^−^ at *m/z* 133 (C_4_H_6_O_5_). It should be noted that citric acid is dominant in citrus peels [36].

Two fatty acids were also annotated and identified as hydroxy-hexadecanoic acid (C_16_H_32_O_3_) and linolenic acid (C_18_H_30_O_2_).

Organic acids, including malic and citric acids, have been widely used as food preservatives because they exhibit a broad spectrum of action against Gram-positive bacteria, Gram-negative bacteria, fungi, and yeasts [37,38].

Identification of Phenolic Acids and Phenolic Acid Conjugates

**Figure 3:**
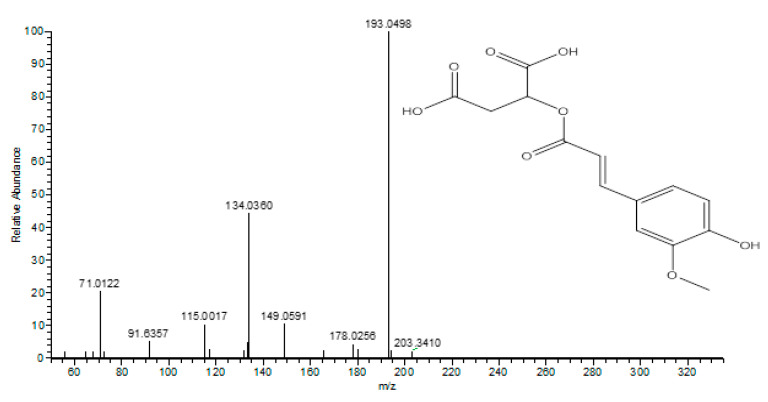
UPLC/MS/MS of feruloyl-*O*-malic acid ester.

Two phenolic acids were annotated as caffeic acid (compound 8) and syringic acid (compound 16). These compounds were accompanied by the loss of [M − H − 44]^−^, characteristic of the loss of a carboxyl group. Compound 7 with [M-H]^-^ at *m/z* 329 and the daughter ion at 167 [M − H − 162]^−^ was assigned as vanillic acid hexoside. Compound 7 with [M − H]^−^ at *m/z* 367 and the daughter ion at 193 [M − H − 191]^−^ was assigned as feruloyl quinic acid. Compound 10 with [M − H]^−^ at *m/z* 399 and the daughter ion at 223 [M − H − 176]^−^ was assigned as sinapic acid hexuronide, which was identified for the first time from mandarin peels, and compound 12 with [M − H]^−^ at *m/z* 385 and the daughter ion at *m/z* 223 [M − H − 162]^−^ was assigned as sinapic acid hexoside. Sinapic acid derivatives were reported before from citrus species [39]. Compound 19, as shown in Figure 3, with [M − H]^−^ at *m/z* 309 and the daughter ion at *m/z* 193 [M − H − 116]^−^ was attributed to ferulic acid, and that at *m/z* 134 and 133 was attributed to the presence of a malic acid moiety. Therefore, compound 19 was identified as feruloyl-*O*-malic acid ester, which was tentatively identified for the first time from mandarin peels.

Moreover, cinnamic acid derivatives from plant material and their conjugates reported having antibacterial and antifungal properties [40].

Identification of Flavonoid-*O*-Glycosides

Flavonoid-*O*-glycosides in general, and especially hesperidin, are, in addition to sugars, the most abundant compounds in mandarin peels. In MS/MS fragmentation spectra, the nature of the sugars in *O*-glycosides could be distinguished from the elimination of the sugar residue from molecular ions, i.e., 162 dalton (glucose or galactose), and 146 dalton (deoxyhexose such as rhamnose) [26]. Therefore, six flavonoid-*O*-glycoside compounds (18, 20, 21, 23, 24, and 25) were annotated, according to GNPS [19,26], to naringenin-7-*O*-rutinoside (C_27_H_32_O_14_), hesperidin (C_28_H_34_O_15_), isosakuranetin-7-*O*-neohesperidoside (C_28_H_34_O_14_), naringenin-7-*O*-hexoside (C_21_H_22_O_10_), rhiofolin (C_27_H_30_O_14_), and isorhamnetin-3-*O*-hexoside (C_22_H_22_O_12_), respectively.

Identification of Flavonoid-C-Glycosides

The fragmentation patterns of C-glycoside flavonoids are [M − H − 120]^−^ and [M − H − 90]^–^ and were clearly observed in compounds **11**, 14, 16, 17, and 22. These compounds were, respectively, identified as apigenin-di-C-hexoside (vicenin 2), methoxyluteolin di-C-hexoside (lucenin-2 4′-methyl ether), orientin, vitexin, and diosmetin-C-hexoside. It should be noted that both luteolin-C-glycosides have been reported in citrus peels [41].

Identification of Polymethoxyflavones and Flavonoid Aglycones

It should be noted that polymethylated flavones have been previously reported in citrus species [42]. In the MS/MS fragmentation spectrum of mandarin peel extract, polymethylated flavones were observed and have distinctive fragmentation patterns including [M − H − nCH_3_] ^−^ and [M − H − nCH_3_ − CO]^−^. These compounds are 28, 29, 30, and 31, identified as dihydroxy-trimethoxyflavone, dihydroxy-dimethoxyflavone, dihydroxy-tetramethoxyflavone, and dihydroxy-dimethoxyflavone isomer, respectively.

Identification of Coumarins

Only one compound, namely, meranzin hydrate, was detected and identified according to its molecular formula and fragmentation pattern.

Identification of Hesperidin by NMR Spectroscopy

An off-white powder of the isolated compound was obtained from mandarin peels, showing molecular ion [M − H]^−^ at *m*/*z* 609 and accompanied by a daughter molecular ion at m/z 301. The isolated compound was further investigated by ^1^H and ^13^C NMR spectroscopy as follows:

^1^H NMR (400 MHz, DMSO-*d*_6_), δ 6.93 (m, 3H, H-2′, H-5′ and H-6′), 6.15 (brs, 1H, H-6), 6.13 (brs, 1H, H-8), 5.50 (dd, *J* = 12.3, 3.3 Hz, 1H, H-2), 2.78 (dd, *J* = 3.2 Hz & 17.15, 1H, H-3_ax_), 3.25 (dd, *J* = 17.16, 8.21 Hz, 1H, H-3_eq_), 4.98 (d, *J* = 7.1 Hz, 1H, H-1″), 4.54 (s, 1H, H-1‴), 1.10 (d, *J* = 6.1, 3H, CH_3_ of rhamnose). ^13^C NMR (101 MHz, DMSO) δ 197.46 (C-4), 165.59 (C-7), 163.50 (C-5), 162.95 (C-9), 148.42 (C-3′), 146.90 (C-4′), 131.34 (C-1′), 118.44 (C-2′), 114.60 (C-6′), 112.48 (C-5′), 103.80 (C-10), 101.05 (C-1‴), 99.92 (C-1″), 96.87(C-6), 96.03 (C-8), 78.83 (C-2), 76.73 (C-3″), 75.99 (C-5″), 73.45 (C-2″), 72.55 (C-4‴), 71.19 (C-2‴), 70.74 (C-3‴), 70.07 (C-4″), 68.79 (C-5‴), 66.50 (C-6″), 56.15 (O-Me), 42.49 (C-3), 18.29 (C-6‴) [28,43].

### 2.3. Antibacterial Activity of NaNO_2_, Mandarin Peel Extract, and Hesperidin

The antibacterial activities of sodium nitrite, mandarin peel extract, and hesperidin against four strains of foodborne pathogenic bacteria are presented in Table 3. Mandarin peel extract exhibited the highest activity against *E. coli*, *P. aeruginosa*, and *B. cereus*, with inhibition zones of 16.7, 13.0, and 10.2 mm, respectively. Additionally, the highest antibacterial activity of hesperidin was recorded against Gram-negative *E. coli*, *P. aeruginosa*, and *P. aeruginosa*, with 15.8 and 10.8 mm inhibition zones, while the highest zone of inhibition of 19.3 mm by sodium nitrite was observed against *P. aeruginosa*. Previously published data confirmed the antimicrobial activity of mandarin peel extract as limonene, the major volatile constituent [44], hesperidin flavonoid [45], and citric acid [46].

#### 2.3.1. Minimum Inhibitory Concentration and Synergy Interactions of Mandarin Peel Extract with NaNO_2_

As illustrated in Table 4, the MIC values obtained from sodium nitrite with mandarin peel extract against the tested pathogenic bacteria ranged between 0.77 and 1.13 mg mL^−1^. The lowest MIC value, 0.77 mg mL^−1^, was recorded against *E*. *coli*, while the highest MIC was observed against *Staph. aureus*, 1.13 mg mL^−1^. MIC values of sodium nitrite ranged from 0.67 to 1.67 mg mL^−1^. All combinations between mandarin peel extract and sodium nitrite were tested against all the described pathogenic strains. As shown in Table 4, a significant decrease in MIC values of sodium nitrite, between 75% and 87.5%, was recorded when combined with mandarin peel extract, this reduction depending upon the tested bacterial strain. Additionally, there was a significant decrease in the MICs of mandarin peel extract when combined with sodium nitrite.

The fraction inhibitory concentration index (FICI) values obtained by the checkerboard assay were in the range of 0.18 to 0.5, indicating that all combinations studied had a synergistic effect (FICI ˂ 0.5) in all tested strains. Mandarin peel extract enhanced the antibacterial activity of sodium nitrite against *B*. *cereus*, *Staph*. *aureus*, *E*. *coli*, and *P*. *aeruginosa* with a synergistic effect (FICI values of 0.37, 0.18, 0.25, and 0.50, respectively).

#### 2.3.2. Minimum Inhibitory Concentration and Synergy Interactions of Hesperidin

The MIC value of hesperidin alone and in combination with sodium nitrate was determined, as shown in Table 5. The MICs of hesperidin and sodium nitrite against the tested pathogenic bacteria varied between 0.67 and 4.76 mg mL^−1^. The lowest MIC value exhibited by hesperidin was against *E*. *coli* (1.13 mg mL^−1^), while the highest MIC observed was 1.53 mg mL^−1^ against *Staph. aureus*. Strong synergistic activity for the combination of hesperidin and sodium nitrite was shown against *B*. *cereus* and *P. aeruginosa* (Table 5). A significant reduction in MICs of hesperidin and sodium nitrite was observed against the tested bacteria. Fraction inhibitory concentration indices (FICIs) showed synergy of 0.37 with *B*. *cereus* and *P. aeruginosa*, and an additive interaction against *Staph*. *aureus* and *E*. *coli* with FICI values of 0.63 and 0.75, respectively. No antagonism was recorded from the hesperidin and sodium nitrite combination.

### 2.4. Time–Kill Assay

**Figure 4:**
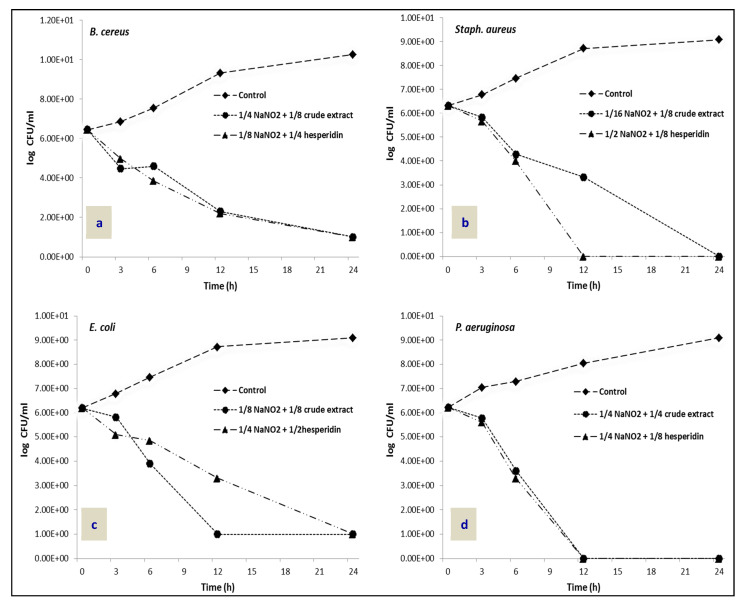
Time–kill data for synergistic combination of NaNO_2_ with mandarin peel extract and hesperidin against (**a**): *B. cereus*; (**b**): *Staph. aureus*; (**c**): *E. coli*; (**d**): *P. aeruginosa.*

To confirm the synergistic effect of mandarin peel extract and hesperidin with NaNO_2_ against the tested foodborne pathogenic bacteria, a time–kill assay was conducted (Figure 4). The combination of 1/4 and 1/8 MICs of NaNO_2_ with 1/8 and 1/4 of mandarin peel extract and hesperidin, respectively, showed effective inhibition against *B. cereus* within 24 h (Figure 4a). Meanwhile, the combination of 1/16 MIC of NaNO_2_ and 1/8 MIC of mandarin peel extract completely inhibited *Staph. aureus* growth within 24 h, and the combination of 1/2 MIC of NaNO_2_ and 1/8 MIC of hesperidin completely inhibited the growth of *Staph. aureus* within 12 h (Figure 4b). The combination of 1/8 and 1/4 MICs of NaNO_2_ with 1/8 and 1/2 of mandarin peel extract and hesperidin, respectively, showed significant inhibition against *E. coli* within 24 h (Figure 4c). Additionally, the combination of 1/4 MIC of NaNO_2_ with 1/4 MIC of mandarin peel extract and 1/8 MIC of hesperidin required only 12 h to completely inhibit the growth of *P*. *aeruginosa* (Figure 4d).

## 3. Materials and Methods

### 3.1. Plant Materials and Extraction

The mandarin fruits were collected from the National Research Center farm, Nubaria, Egypt.

The fresh mandarin peels (1000 g) were mixed with ethanol in a blender (each 100 g:1 L of ethanol) three times, followed by filtration and evaporation at 40 °C to yield sticky material (28 g) of the total ethanolic extract.

### 3.2. Gas Chromatography-Mass Spectrometry Analysis (GC/MS)

To prepare a sample for GC/MS analysis, 50 g of fresh mandarin peels was mixed in a blender with 500 mL ethanol, then the filtrate was collected, and 50 mL of water was added to the filtrate. Hence, the filtrate was partitioned with hexane according to the method described in nature protocols by Kjer et al., 2010 [47]. The n-hexane fraction was evaporated under vacuum using a rotatory evaporator at 35 °C for 25 min to yield a volatile oil.

The n-hexane fraction was applied to the GC/MS system (Agilent Technologies, Santa Clara, CA 95051. USA) equipped with a gas chromatograph (7890B) and mass spectrometer detector (5977A) at Central Laboratories Network, National Research Centre, Cairo, Egypt. The GC was also equipped with an HP-5MS column (30 m × 0.25 mm internal diameter and 0.25 μm film thickness). Helium was used as a carrier gas at a flow rate of 1.0 mL/min at a split of 1:30, injection volume of 1 µL, and the following temperature program: 40 °C for 1 min; rising at 4 °C /min to 150 °C and holding for 6 min; rising at 4 °C/min to 210 °C and holding for 1 min. The injector and detector were held at 280 °C and 220 °C, respectively. Mass spectra were obtained by electron ionization (EI) at 70 eV, using a spectral range of *m*/*z* 50–900 and solvent delay of 5 min. Wiley and NIST Mass Spectral Library data were used for identification of mandarin peel volatile n-hexane fraction constituents by comparing the spectrum fragmentation pattern with those stored in the data.

### 3.3. Ultra-Performance Liquid Chromatography-Mass Spectrometry Analysis (UPLC/MS/MS)

UPLC/MS/MS analysis was performed according to the method described in Ammar et al., 2021 [48], in which the UPLC model, Waters (Milford, Massachusetts, MA 01757. USA) hyphenated to the Q Exactive hybrid MS/MS quadrupole - Orbitrap mass spectrometer was used. Chromatographic separation for this system was carried out using acidified water with 0.1% formic acid (solvent 1) and acetonitrile (solvent 2) with a mobile phase flow rate of 0.4 mL/min in the following gradient: 0–15 min from 50% to 50% solvent 2; 15–22 min to 98% of solvent 2, maintaining these conditions for 22 min; then, from 22 to 23 min 95% of solvent 1 to 27 min system, returning to starting conditions and re-equilibrating for 3 min with the BEH shield C18 column (150 × 2.1 mm, 1.7 μm). The Q-Exactive MS operated upon the following settings: HESI ion source voltage −3 kV or 3 kV; sheath gas (N2) flow 48 L/min; auxiliary gas flow 13 L/min; ion source capillary temperature 250 °C; auxiliary gas heater temperature 38 °C. The CID MS/MS experiments were performed using a collision energy of 15 eV.

### 3.4. Isolation and Identification of Hesperidin

Mandarin peel extract (25 g) was applied to polyamide 6 (Sigma-Aldrich, Munich, Germany) column chromatography (250 g), eluted by water to yield 12.5 g of sticky material, mainly sugars, and then eluted with 30% ethanol in order of decreasing polarity to yield 4.2 g of yellow amorphous powder, then 60% ethanol to yield 3.3 g, and finally 100% ethanol 2.1g after evaporation of the eluent under vacuum using a Heidolph rotatory evaporator (Schwabach, Germany).

The presence of hesperidin was checked using comparative paper chromatography (Whatman filter paper sheets No.1, United Kingdom) using 15% acetic acid as aqueous eluent and butanol/acetic acid/water (BAW) in portions (4:1:5, respectively) as organic eluent. The 30% ethanol fraction (4 g) was applied to Sephadex LH-20 column chromatography (Pharmacia Company, Uppsala, Sweden) and eluted with 50% ethanol and monitored by UV lamp (365 nm). Then, the collected fractions were checked by comparative paper chromatography using hesperidin as a standard sample (friendly, 2 mg obtained from the Department of Phytochemistry and Plant Systematics, National Research Center, Dokki, Cairo, Egypt), whereby 750 mg of hesperidin-containing sub-fractions was collected. The hesperidin sub-fraction was applied to repeated Sephadex LH-20 column chromatography using butanol saturated with water as eluent to finally yield 400 mg of hesperidin.

### 3.5. Nuclear Magnetic Resonance (NMR Analysis)

^1^H and ^13^C-NMR spectra were acquired using an Avance III HD 400 MHz NMR spectrometer (Bruker BioSpin GmbH, Rheinstetten, Germany). NMR spectra of the isolated compound were measured in DMSO-*d*_6_ and calibrated to the residual solvent signals resonances at δ H = 2.49 and δ C = 39.5 ppm [49].

### 3.6. Antibacterial Activity Assay

#### 3.6.1. Tested Bacteria Strains

The antibacterial activity of sodium nitrite, mandarin peel extract, and hesperidin was tested on two Gram-positive bacteria (*Staphylococcus aureus* (ATCC 25923) and *Bacillus cereus* (EMCC 1080)), and two Gram-negative bacteria (*Pseudomonas aeruginosa* (NRRL B-272) and *Escherichia coli* (0157 H7 ATCC 51659)). The antibacterial assays were obtained from VACSERA (Holding Company for Biological Products and Vaccines), Egypt. The stock cultures were grown on slants of nutrient agar at 37 °C for 24 h and then kept in the refrigerator.

#### 3.6.2. Disc Diffusion Technique

A loop full of bacteria, incubated for 24 h in a nutrient agar slant of each bacterial species, was inoculated in a test tube containing 5 mL of tryptic soy broth. Broth culture was incubated at 37 °C for 4 h until it achieved turbidity of 0.5 McFarland BaSO_4_ standard (10^8^ cfu mL^−1^). The sensitivity tests of mandarin peel extract, hesperidin, and sodium nitrite were determined with different bacterial strains using the disc diffusion method by the Kirby–Bauer technique [50,51]. DMSO represented the negative control. After that, inoculated plates were incubated at 37 °C for 24 h. At the end of the incubation period, inhibition zones were expressed as the diameter of clear inhibition zone including the diameter of the paper disc.

### 3.7. Determination of Minimum Inhibitory Concentration (MIC)

Minimal inhibitory concentrations (MICs) for mandarin peel extract, hesperidin, and sodium nitrite were determined using the microbroth dilution method by Andrews et al., 2001 [52]. Two-fold serial dilutions of mandarin peel extract, hesperidin, and sodium nitrite ranging from 10 to 0.05 mg mL^−1^ were used. Equal volumes of tested bacteria (10^5^ cfu mL^−1^) were added to each well. MIC values were taken as the lowest concentration of the antimicrobial agent that inhibited bacterial growth after 24 h of incubation at 37 °C.

### 3.8. Checkerboard Assay

The presence of synergism or antagonism of mandarin peel extract and hesperidin with NaNO_2_ was evaluated using isobolograph analyses and the checkerboard assay according to [53,54]. This method was conducted using different concentrations of samples and sodium nitrite along different axes, ensuring that each well contained different combinations of the samples and sodium nitrite. The analyses were performed using 96-well plates. Bacteria were grown to reach 2 × 10^8^ cfu mL^−1^. Five microliters of each bacterial strain inoculum was added into the well containing tested samples, sodium nitrite, and Mueller Hinton Broth medium (MHB). The plates were incubated for 18 h/37 °C. MIC was determined for the combination as the lowest concentration that completely inhibited bacterial growth. Fractional inhibitory concentration (FIC) was calculated for each combination using the following formula: FICA = MICA in combination/MICA alone; FICB = MICB in combination/MICB alone; FIC index = FICA + FICB, where MICA is the MIC of NaNO_2_, FICA is the FIC of NaNO_2_, MICB is the MIC of mandarin peel extract or hesperidin, and FICB is the FIC of the mandarin peel extract or hesperidin. FIC index is the FIC added value of both NaNO_2_ and mandarin peel extract or hesperidin. The interaction of the antibacterial combinations was determined as previously reported by [55,56,57] by plotting an isobologram.

### 3.9. Time–Kill Assay

Time–kill curves were assayed using the confirmed synergistic combinations of mandarin peel extract or hesperidin with NaNO_2_ against tested bacteria. The overnight growth plate was inoculated in sterile MHB at 35 °C to approximate the density of 0.5 McFarland standard. The suspension was diluted 1:10 in normal saline solution to obtain a standard inoculum of 1 × 10^6^ CFU/mL. An amount of 100 μL of the diluted bacterial suspension was added to 0.9 mL of MHB. Double dilutions for each NaNO_2_ and sample were prepared. Tubes containing individual NaNO_2_ and the combination were incubated at 35 °C for 24 h. From each tube, 100 μL of the sample was collected at 0, 3, 6, 12, and 24 h and plated to determine the count of viable cells. Additionally, growth control was included for each assay. The killing rate was determined by plotting colony viable counts (CFU/mL) against time. Synergy was defined as a ≥ 2 log_10_ CFU/mL reduction in viable bacteria with the combination compared with the most active single agent.

## 4. Conclusions

There is no escaping that food preservatives should be used to increase the shelf life of food products and to avoid the economical loss that can arise as a result of food spoilage. Additionally, there is no doubt that there are hazards of using these chemical preservatives to human health. Therefore, the solution is either finding a safe and natural substitute, which is actually tedious and costly, or decreasing the harm of these preservatives by maximizing their preservative effect at low concentrations. This work provides a solution by proving the synergistic and/or additive action of a natural by-product and it’s bioactive flavonoid (hesperidin) against selected food pathogen microbes. This study showed a significant decrease in the MIC values of sodium nitrite with mandarin peel extract, ranging between a 75% to 87.5% reduction depending on the tested strain; further, hesperidin showed both synergistic and additive activities. The application of any of them as a preservative is related to the quality measures, e.g., taste, odor, and homogeneity, that need to be achieved in food products.

## Figures and Tables

**Table 1:** Identified volatile compounds by GC/MS spectrometry.

Peak	RT	Name	Formula	Area	Area Sum %
1	7.016	l-Phellandrene	C_10_H_16_	18,336,447	0.47
2	7.205	α-(-)-pinene	C_10_H_16_	53,171,439.3	1.37
3	8.453	3-Carene	C_10_H_16_	5,287,840.96	0.14
4	8.519	2-.β-pinene	C_10_H_16_	44,154,069.3	1.13
5	9.096	β-myrcene	C_10_H_16_	59,501,702.7	1.53
6	9.9	2-Carene	C_10_H_16_	11,948,052.3	0.31
7	10.628	D-Limonene	C_10_H_16_	2,928,075,732	75.21
8	11.488	ɤ-Terpinene	C_10_H_16_	725,626,383	18.64
9	12.33	α-Terpinolene	C_10_H_16_	30,695,286.9	0.79
10	21.767	α-ylangene	C_15_H_24_	2,130,866.43	0.05
11	23.081	Caryophyllene	C_15_H_24_	8,035,838.48	0.21
12	25.369	1H-Cycloprop[e]azulene, 1a,2,3,5,6,7,7a,7b-octahydro-1,1,4,7-tetramethyl-, [1aR-(1a.alpha.,7.alpha.,7a.beta.,7b.alpha.)]-	C_15_H_24_	3,360,910.92	0.09
13	26.211	β-copaene	C_15_H_24_	3,051,328.78	0.08

**Table 2:** UPLC/MS/MS of mandarin peels.

No	RT [Min]	Metabolite Identification	Chemical Formula	[M − H]^−^		Ref.
				Measured	Fragmentation	
1	2.03	Trehalose	C_12_H_22_O_11_	341.1092	179.0552	[19]
2	2.08	Citric acid	C_7_H_11_O_6_	191.0552	173.0445	[19]
3	2.15	Hexose	C_6_H_12_O_6_	179.0550	161.0443	[19]
4	2.16	Malic acid	C_4_H_6_O_5_	133.0128	115.0022	[20]
6	3.21	Tryptophan	C_11_H_12_N_2_O_2_	203.0818	186.0546, 159.0915, 142.0650, 116.0491	[21]
7	3.39	Vanillic acid hexoside	C_14_H_18_O_9_	329.0879	167.0337	[22]
8	3.41	Caffeic acid	C_9_H_8_O_4_	179.0551	134.9866	[23]
9	4.25	Feruloyl quinic acid	C_17_H_20_O_9_	367.1033	193.0497, 191.0185	[24]
10	4.29	Sinapic acid hexouronide	C_17_H_20_O_11_	399.0932	223.0462, 193.0497	[25]
11	4.46	Apigenin-di-C-hexoside (vicenin 2)	C_27_H_30_O_15_	593.1360	503.1203, 473.1094, 383.0774, 353.0667	[26]
12	4.48	Sinapic acid hexoside	C_17_H_22_O_10_	385.1854	223.1331	[27]
13	4.73	Meranzin hydrate	C_15_H_18_O_5_	277.1080	259.0951, 233.1181, 215.1074, 189.9480, 87.0070	[28]
14	4.75	Methoxyluteolin di-C-hexoside	C_30_H_38_O_16_	623.1752	533.1318, 503.1197, 413.6878, 383.0773	[26]
15	4.87	Syringic acid	C_9_ H_10_ O_5_	197.0446	169.0130, 125.0227	[22]
16	4.89	Orientin	C_21_H_20_O_11_	447.0935	357.0616, 327.0511	[19]
17	5.28	Vitexin	C_21_H_20_O_10_	431.0985	341.0668, 311.0562	[29]
18	5.47	Naringenin-7-*O*-rutinoside	C_27_H_32_O_14_	579.1774	271.0612	[19]
19	5.48	Feruloyl-*O*-malic acid ester	C_14_H_13_O_8_	309.0616	193.0498, 134.0360, 115.0017	[30]
20	5.55	Hesperidin	C_28_H_34_O_15_	609.1814	475.2522, 430.9161, 367.2440, 301.0651	[31]
21	5.52	Isosakuranetin-7-*O*-neohesperidoside	C_28_H_34_O_14_	593.1551	285.0405	[26]
22	5.59	Diosmetin-C-hexoside	C_22_H_22_O_11_	461.1085	371.0770, 341.0664, 298.0481	[32]
23	5.68	Naringenin-7-*O*-Hexoside	C_21_H_22_O_10_	433.1142	271.0614	[26]
24	5.79	Rhiofolin	C_27_H_29_O_14_	577.0258	269.0456	[33]
25	5.88	Isorhamnetin-3-*O*-hexoside	C_22_H_22_O_12_	477.1044	315.0505, 314.0432, 300.0292	[34]
26	7.41	Diosmetin	C_16_H_12_O_6_	299.0550	284.0326	[19]
27	7.51	hesperitin	C_16_H_14_O_6_	301.0618	286.0382	[19]
28	8.04	Dihydroxy trimethoxy flavone	C_18_H_15_O_7_	343.0823	328.0589, 313.0355, 298.0126, 285.0407	[26]
29	8.10	Dihydroxy dimethoxy flavone	C_17_H_13_O_7_	329.0328	314.0423, 300.509, 299.0182	[26]
30	8.23	Dihydroxy tetramethoxy flavone	C_19_H_18_O_8_	373.0932	358.0694, 343.0458, 328.0222, 300.0268	[26]
31	8.25	Dihydroxy trimethoxy flavone	C_18_H_15_O_7_	343.0819	328.0589, 313.0355, 298.0126, 285.0407	[26]
32	9.12	Hydroxy-hexadecanoic acid	C_16_H_31_O_3_	271.1914	253.1805, 209.1901	[26]
33	10.25	Linolenic acid	C_18_H_30_O_2_	277.2170	233.1541, 205.1590, 59.0121	[31]

**Table 3:** Antibacterial activity of mandarin peel extract, hesperidin, and sodium nitrite against some foodborne pathogenic bacteria.

Bacteria	Inhibition Zone, mm (Mean ± S.E)			
	Negative Control	NaNO_2_ 1 mg mL^−1^	Mandarin Peel Extract 10 mg mL^−1^	Hesperidin 10 mg mL^−1^
***B. cereus***	0	15.2 ± 1.04 ^a^	10.2 ± 0.81 ^b^	8.8 ± 0.76 ^c^
***Staph. aureus***	0	17.2 ± 0.76 ^a^	9.3 ± 0.58 ^b^	9.8 ± 1.25 ^b^
***E. coli***	0	11.8 ± 0.76 ^c^	16.7 ± 2.46 ^a^	15.8 ± 0.86 ^b^
***P. aeruginosa***	0	19.3 ± 1.04 ^a^	13.0 ± 0.50 ^b^	10.8 ± 0.86 ^c^

*n* = 3, *p* ˂ 0.05, S.E: standard error; DMSO: negative control. Values are given as mean ± SE. Means followed by different superscripts within rows (a, b, and c) are significantly different.

**Table 4:** Synergic interaction between mandarin peel extract with NaNO_2_ against the tested foodborne pathogenic bacteria.

Bacteria	MICA mg mL^−1^	MICB mg mL^−1^	FICA	FICB	FIC Index	Interaction
***B. cereus***	1.67	0.93	0.25	0.12	0.37	S
***Staph. aureus***	0.92	1.13	0.06	0.12	0.18	S
***E. coli***	4.76	0.77	0.13	0.12	0.25	S
***P. aeruginosa***	0.67	1.03	0.25	0.25	0.5	S

*n* = 3, MICA: MIC of NaNO_2_; MICB: MIC of mandarin peel extract; FICA: FIC of NaNO_2_; FICB: FIC of mandarin peel extract; S: synergistic effect, if ∑FIC index ≤ 0.5.

**Table 5:** Synergic interaction between hesperidin and NaNO_2_ against the tested foodborne pathogenic bacteria.

Bacteria	MICA mg mL^−1^	MICB mg mL^−1^	FICA	FICB	FIC Index	Interaction
***B. cereus***	1.67	1.33	0.13	0.25	0.37	S
***Staph. aureus***	0.92	1.53	0.5	0.13	0.63	A
***E. coli***	4.76	1.13	0.25	0.5	0.75	A
***P. aeruginosa***	0.67	1.27	0.25	0.13	0.37	S

*n* = 3, *p* ˂ 0.05, MICA: MIC of NaNO_2_; MICB: MIC of hesperidin; FICA: FIC of NaNO_2_; FICB: FIC of hesperidin; S: synergistic effect if ∑FIC ≤ 0.5; A: additive if 0.5 < ∑FIC ≤ 1.

## Data Availability

Data are contained within the article.

## Sample Availability

Samples of the compounds are available from the authors.
